# Evaluation of the Clinical Efficacy and Trust in AI-Assisted Embryo Ranking: Survey-Based Prospective Study

**DOI:** 10.2196/52637

**Published:** 2024-06-03

**Authors:** Hyung Min Kim, Hyoeun Kang, Chaeyoon Lee, Jong Hyuk Park, Mi Kyung Chung, Miran Kim, Na Young Kim, Hye Jun Lee

**Affiliations:** 1 AI Lab Kai Health Seoul Republic of Korea; 2 IVF Clinic Miraewaheemang Hospital Seoul Republic of Korea; 3 IVF Clinic Seoul Rachel Fertility Center Seoul Republic of Korea; 4 Department of Obstetrics & Gynecology Ajou University School of Medicine Suwon Republic of Korea; 5 IVF Clinic HI Fertility Center Seoul Republic of Korea; 6 Kai Health Seoul Republic of Korea

**Keywords:** assisted reproductive technology, in vitro fertilization, artificial intelligence, intraobserver and interobserver agreements, embryos, embryologists

## Abstract

**Background:**

Current embryo assessment methods for in vitro fertilization depend on subjective morphological assessments. Recently, artificial intelligence (AI) has emerged as a promising tool for embryo assessment; however, its clinical efficacy and trustworthiness remain unproven. Simulation studies may provide additional evidence, provided that they are meticulously designed to mitigate bias and variance.

**Objective:**

The primary objective of this study was to evaluate the benefits of an AI model for predicting clinical pregnancy through well-designed simulations. The secondary objective was to identify the characteristics of and potential bias in the subgroups of embryologists with varying degrees of experience.

**Methods:**

This simulation study involved a questionnaire-based survey conducted on 61 embryologists with varying levels of experience from 12 in vitro fertilization clinics. The survey was conducted via Google Forms (Google Inc) in three phases: (1) phase 1, an initial assessment (December 23, 2022, to January 22, 2023); (2) phase 2, a validation assessment (March 6, 2023, to April 5, 2023); and (3) phase 3 an AI-guided assessment (March 6, 2023, to April 5, 2023). Inter- and intraobserver assessments and the accuracy of embryo selection from 360 day-5 embryos before and after AI guidance were analyzed for all embryologists and subgroups of senior and junior embryologists.

**Results:**

With AI guidance, the interobserver agreement increased from 0.355 to 0.527 and from 0.440 to 0.524 for junior and senior embryologists, respectively, thus reaching similar levels of agreement. In a test of accurate embryo selection with 90 questions, the numbers of correct responses by the embryologists only, embryologists with AI guidance, and AI only were 34 (38%), 45 (50%), and 59 (66%), respectively. Without AI, the average score (accuracy) of the junior group was 33.516 (37%), while that of the senior group was 35.967 (40%), with *P*<.001 in the *t* test. With AI guidance, the average score (accuracy) of the junior group increased to 46.581 (52%), reaching a level similar to that of the senior embryologists of 44.833 (50%), with *P*=.34. Junior embryologists had a higher level of trust in the AI score.

**Conclusions:**

This study demonstrates the potential benefits of AI in selecting embryos with high chances of pregnancy, particularly for embryologists with 5 years or less of experience, possibly due to their trust in AI. Thus, using AI as an auxiliary tool in clinical practice has the potential to improve embryo assessment and increase the probability of a successful pregnancy.

## Introduction

Infertility affects 1 in 6 couples worldwide, making in vitro fertilization (IVF) a widely sought-after solution. However, the success rate of IVF remains relatively low, typically ranging from 20% to 30% [1,2]. Amidst the ongoing efforts to improve IVF outcomes, the paramount challenge lies in selecting the most viable embryo for transfer, as embryo quality is critical for a successful outcome.

Traditionally, embryologists rely on morphological assessment for selecting embryos [3-5], which involves the observation of embryos under a microscope and assigning grades based on criteria, such as blastocyst expansion stage, inner cell mass, and trophectoderm development. Some laboratories select euploid embryos through preimplantation genetic testing [6]. Even after the preimplantation genetic testing, morphological assessment remains crucial to selecting the most viable one from multiple euploid embryos.

However, this reliance on morphological assessment raises concerns because of its inherent subjectivity and the substantial variability observed in both intra- and interobserver assessments [7-11]. This variability underscores the pressing need for standardized methods of embryo evaluation across laboratories and the IVF industry.

Recent advancements have introduced artificial intelligence (AI) as a complementary tool for morphological evaluation of embryos. By leveraging deep learning techniques, AI-based systems can predict IVF outcomes by learning from extensive sets of embryo images, thus reducing human bias and potentially providing more objective and accurate results [12-17]. Several studies have reported significant advantages of AI-selected embryos in terms of pregnancy rates, compared to embryos chosen through traditional morphological assessment by embryologists [12,15,18].

Despite the notable progress in research on embryo selection using AI, its widespread adoption hinges on proving its clinical efficacy and securing the trust of clinicians. However, demonstrating the clinical efficacy of AI in a real-world setting poses challenges, as pregnancy outcomes can only be observed for selected embryos and not for those left unselected. Furthermore, the ultimate decision regarding which embryos to transfer rests squarely with clinicians, who may either embrace or question AI recommendations. In cases where clinicians opt not to trust AI, the resulting IVF outcomes may not accurately reflect the precision of the AI model’s predictions.

Previous attempts to address these critical questions have involved simulation studies that compared the accuracy of and pregnancy rates facilitated by AI-driven embryo selection and assessments by embryologists [19,20]. However, these studies predominantly relied on historical data from embryo grading records provided by embryologists from various laboratories. This retrospective approach inadvertently introduced substantial sources of variability. Intra- and interobserver agreements among embryologists, which arise from the differing standards and criteria for embryo evaluation, played a significant role in shaping the results. Moreover, the lack of standardization of embryo evaluation across laboratories further complicates the interpretation of the findings.

In addition to these challenges, previous simulation studies have not examined the nuanced demographic profiles and attitudes of embryologists themselves. Understanding these factors can shed light on the broader dynamics at play and offer insights into how AI may be influenced by the unique characteristics and perspectives of the medical professionals involved.

In this study, we aimed to evaluate the clinical efficacy of AI in embryo selection by simulating a clinical setting in which embryologists ranked the embryos for transfer. We assessed the intra- and interobserver agreements of the embryologists’ evaluations to emphasize the need for alternative embryo selection methods. Moreover, we compared the accuracy of embryologists with and without AI assistance, as well as AI-only selection to substantiate the clinical efficacy of AI. Additionally, we analyzed and compared the results for subgroups of embryologists with varying levels of experience to identify distinct practice patterns and levels of trust in AI. We believe that the results of this simulation study can serve as a foundational step for large-scale clinical investigations.

## Methods

### Study Design

This study was a prospective cohort study in which a web-based questionnaire-based survey was conducted among embryologists with varying degrees of experience. The intra- and interobserver agreements of the evaluation of 360 day-5 embryos by the embryologists and the use of a self-developed AI tool were assessed as references for embryologists.

### Ethical Considerations

The embryo images used in our study were collected from 7 IVF clinics in accordance with the Declaration of Helsinki and the institutional review boards (IRBs) of Miraewaheemang Hospital (2022-RESEARCH-01), Good Moonhwa Hospital (GMH-2022-01), the HI Fertility Center (HIRB 2022-01), Seoul Rachel Fertility Center (RTR-2022-01), Ajou University Hospital (AJIRB-MED-MDB-21-716), Pusan National University Hospital (2204-003-113), and Seoul National University Bundang Hospital (B-2208-772-104). Because this study used retrospective data collected through the IRBs of the aforementioned institutions, informed consent was waived, and the personal information contained in the data were deidentified.

### Survey

The survey was conducted in three phases via Google Forms: (1) phase 1, an initial assessment (December 23, 2022, to January 22, 2023); (2) phase 2, a validation assessment (March 6, 2023, to April 5, 2023); and (3) phase 3, an AI-guided assessment (March 6, 2023, to April 5, 2023). The participants received an email with the link to the Google Forms and an attachment of original embryo images included in the questions in case they wanted an enlarged view. The submitted survey results were collected and used for analysis.

To measure intra- and interobserver agreements, the initial assessment and the validation assessment were performed 1 month apart and the questions in the initial assessment were identical to those in the validation assessment. Intraobserver agreement was analyzed based on the differences in responses between the initial and validation assessments (phases 1 and 2), while interobserver agreement was assessed based on the average of the responses. To assess the efficacy of AI guidance, the AI-guided assessment (phase 3) was performed right after the validation assessment.

**Figure 1:**
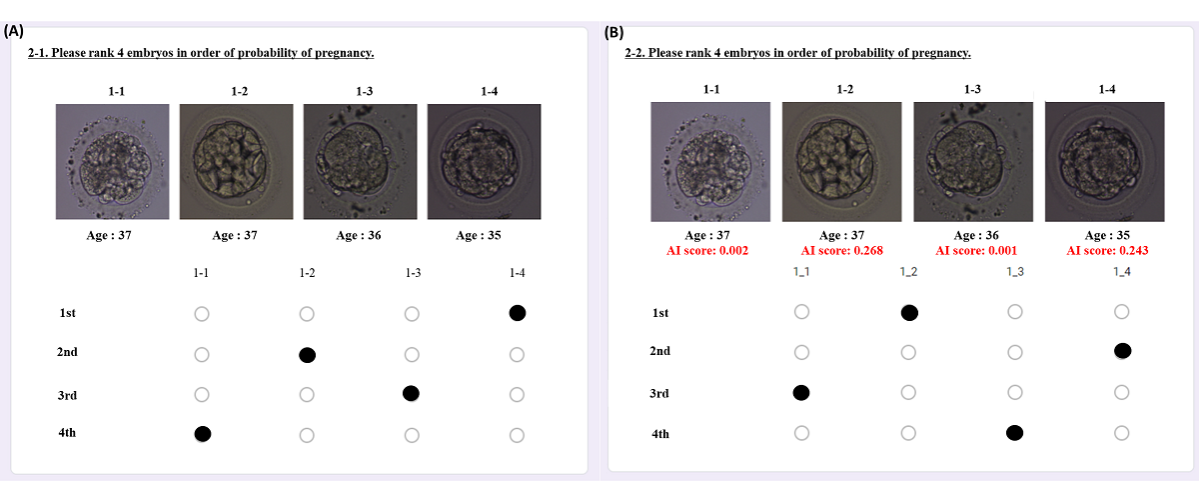
Questions on embryo selection from the web survey. (A) Phase 2 of the survey—question on embryo selection without AI scores. (B) Phase 3 of the survey—question on embryo selection with AI scores. AI: artificial intelligence.

The questionnaire was divided into 2 major sections—items designed to analyze the accuracy of embryo selection and demographic information (age, sex, highest educational level, and tenure). Each assessment consisted of 90 questions and each question consisted of images of 3 embryos that did not result in clinical pregnancy and 1 embryo that did. In the initial and validation assessments, the embryologists were asked to arrange the images in the order of the embryos with the highest likelihood of pregnancy. In the AI-guided assessment, AI scores were provided alongside the embryo images, and the embryologists were asked to reorder the embryos based on their perceived likelihood of pregnancy while considering the AI scores (Figure 1).

Out of 90 questions, 70 questions featured day-5 embryos at the same developmental stage and the remaining 20 questions used randomly selected day-5 embryos regardless of developmental stage to compare the effect of the developmental stage of embryos in embryo selection. To minimize the effect of women’s age, each question contained images of embryos from women of the same age group; younger than 37 years of age and 37 years of age and older.

After the survey, the embryologists were asked to express their opinions on the aspects of (1) the difficulty of the test and the reason for their opinion about the difficulty; (2) the reason for the criteria that they considered when ranking the selected embryos (eg, age, AI score, and image); (3) the reason for changing the answer based on AI score; and (4) consideration when selecting embryos conventionally, as opposed to using a test approach.

### Participants

We conducted an initial assessment with 34 embryologists to build a baseline and included 27 more embryologists for validation and AI-guided assessment. A total of 61 embryologists were recruited from 12 different IVF clinics and had clinical experience of 1 to over 30 years. All participants gave informed consent web-based before participating, and those who refused to give consent were excluded. Among them, 50 Korean embryologists were certified by the Korean Association for Clinical Embryologists and conducted an average of 40-150 cases per month. Of the remaining embryologists, 9 were from Malaysia and 2 were from the United States. These participants were divided into two groups, (1) a junior group consisting of embryologists with embryo grading experience of more than or equal to 5 years and (2) a senior group consisting of embryologists with embryo grading experience of more than 5 years.

**Figure 2:**
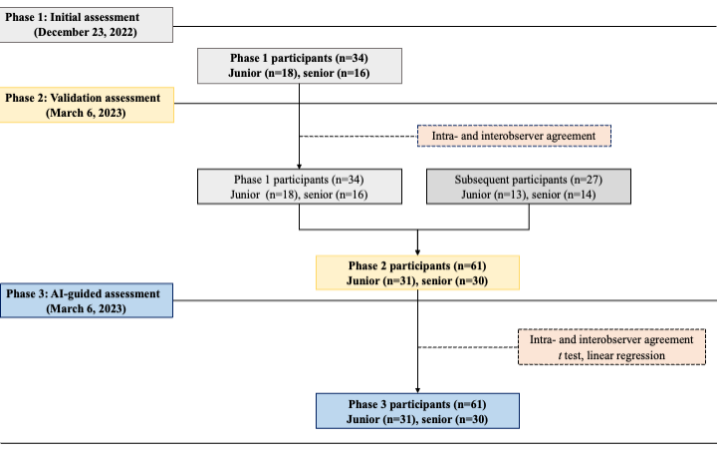
Study design and participant flow diagram according to the survey phase. AI: artificial intelligence.

All 61 participants successfully completed the validation and AI-guided assessments without any loss to follow up. To analyze the results of the validation and AI-guided assessments conducted before and after referring to the AI scores, 29 additional participants were recruited. Statistical analyses were conducted on all participants together and between groups (Figure 2), respectively. From the junior group, 18, 31, and 31 embryologists participated in the initial, validation, and AI-guided assessments, respectively, and from the senior group 16, 30, and 30 embryologists participated in the initial, validation, and AI-guided assessments, respectively.

### Evaluation of the Impact of AI Guidance on Embryo Selection

We measured the accuracy of embryo selection over 4 cycles to determine how much it increased when the embryologists were guided by AI. The first cycle refers to the case in which the embryo selected as rank 1 resulted in clinical pregnancy, and the second cycle refers to the case in which the embryo selected as rank 1 or 2 resulted in clinical pregnancy. Likewise, the third cycle means that the embryo selected as 1, 2, or 3 resulted in clinical pregnancy, and the fourth cycle means 100% because there are 4 examples per question.

### Evaluation of Trust in AI

The model used to infer the AI scores per image was trained on 2555 day-5 embryo images collected from 7 Korean IVF clinics. A total of 2555 images were divided into 2 sets—a training data set consisting of 2043 (80%) images and a model performance test data set containing 512 (20%) images. We then used a 3-fold cross-validation approach to further divide the 2043 training images into 3 separate folds. Each fold was used for both training and validation of the model, and performance was assessed using a fixed model performance test data set. When trained using the ResNet50 architecture, the performance resulted in an area under the receiver operating characteristic of 0.716 and an accuracy of 0.663 [21]. A total of 360 embryo images used in this survey were extracted from 512 images in the data set that were not used to train the AI model. For our questions in the clinical study, the accuracy was 59 (66%), which is similar to the accuracy of the model test set. In assessing the accuracy of the AI model’s predictions, we used a method centered on the alignment of AI scores with the actual clinical outcomes. For each set of embryo images, encompassing 4 images per case, our criterion for determining accuracy was the extent to which the AI’s highest score matched with the embryo led to a successful pregnancy.

To determine the extent to which embryologists relied on AI scores to modify their responses, we defined the AI trust level as follows:



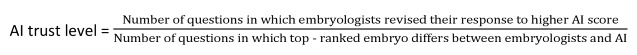



The formula resulted in a trust level of 0-1 for each embryologist. A level of 0 indicates that the embryologist performed all modifications to their responses on a subjective basis with no reference to the AI, whereas a level of 1 indicates that the embryologist performed all modifications so that their ranking was as consistent with the AI score as possible.

### Statistical Analysis

Statistical analyses were performed to assess the consistency of embryo scoring between the embryologists. The Cohen κ coefficient was used to evaluate the intraobserver agreement between the scores given by the same embryologist at 2 different time points [22], whereas Fleiss κ coefficient was used for interobserver agreement between the scores given by different embryologists [23]. The κ coefficient was subsequently construed as excellent (≥0.80), good (0.60-0.79), moderate (0.40-0.59), poor (0.20-0.39), or very poor (<0.20) levels of intra- and interobserver agreements [24]. In this study, we used a 2-tailed *t* test and linear regression to compare the selection accuracy and AI trust level between junior and senior groups. IBM SPSS Statistics (version 29.0; IBM Corporation) was used to quantify intra- and interobserver agreements, while the Python (version 3.8.0; Python Software Foundation) was used to conduct *t* tests and linear regression analyses. When performing the *t* test, as our data consisted of more than 30 samples per group, we assumed a normal distribution based on the central limit theorem.

## Results

### Demographics

The demographics of the 2 groups (Table 1) show that the junior group comprised 25 (81%) female participants and 6 (19%) male participants. The most common age subgroup of the junior group was the 30-39 (n=16, 52%), followed by 20-29 (n=14, 45%) and 40-49 (n=1, 3%). The senior group, on the other hand, had a proportion of 21 (70%) female participants and 9 (30%) male participants, with the largest proportion of embryologists (n=10, 33%) being in their 40s and 50s, followed by those in their 30s (n=9, 30%) and 20s (n=1, 4%). Over half of the junior and senior groups (n=18, 58% and n=15, 50%, respectively) had a master’s degree. The junior group comprised 13 (42%) university graduates and no doctoral degree holders. The senior group had a high percentage of doctoral degree holders (n=10, 33%), followed by bachelor’s degrees (n=5, 17%). In relation to embryo assessment expertise, the junior group had an average of 1.6 years of experience, while the senior group had an average of 13.2 years of experience.

**Table 1:** Demographic characteristics of the junior and senior groups.

Characteristics		Junior	Senior
**Sex, n (%)**			
	Male	6 (19)	9 (30)
	Female	25 (81)	21 (70)
**Age (years), n (%)**			
	20-29	14 (45)	1 (4)
	30-39	16 (52)	9 (30)
	40-49	1 (3)	10 (33)
	≥50	0 (0)	10 (33)
**Highest level of education, n (%)**			
	Bachelor’s degree	13 (42)	5 (17)
	Master’s degree	18 (58)	15 (50)
	Doctoral degree	0 (0)	10 (33)
Experience in embryo selection (years), mean (SD)		1.6 (1.9)	13.2 (7.4)

### Intra- and Interobserver Agreements

The evaluation of intraobserver agreement revealed a Cohen κ score of 0.662 between the initial and validation assessments, indicating good (0.60-0.79) concordance between the responses of 1 embryologist at 2 separate time points. The coefficients for the junior and senior groups were 0.659 and 0.664, respectively, indicating that the less experienced group was able to provide consistent responses at a similar level as the experienced group (Table 2). Additionally, the correlation coefficients between the validation and AI-guided assessments were 0.735 for the overall population and 0.698 and 0.773 for the junior and senior groups, respectively. This indicates that all participants showed improved consistency after the AI guidance.

**Table 2:** Results of the evaluation of the intra- and interobserver agreements.

Characteristics	Intraobserver agreement, Cohen κ coefficient (95% CI)		Interobserver agreement, Fleiss κ coefficient (95% CI)	
	Without AI^a^ (phase 1^b^ vs phase 2^c^)	With AI vs without AI (phase 2 vs phase 3^d^)	Without AI (phase 2)	With AI (phase 3)
Overall	0.662 (0.631-0.692)	0.735 (0.670-0.770)	0.392 (0.389-0.395)	0.521 (0.518-0.524)
Junior	0.659 (0.603-0.714)	0.698 (0.653-0.744)	0.355 (0.349-0.360)	0.527 (0.521-0.532)
Senior	0.664 (0.604-0.710)	0.773 (0.722-0.825)	0.440 (0.434-0.445)	0.524 (0.518-0.529)

^a^AI: artificial intelligence.

^b^Initial assessment.

^c^Validation assessment.

^d^AI-guided assessment

Next, we measured the Fleiss κ coefficient for interobserver agreement between multiple embryologists and found that validation assessment showed a poor coefficient (0.20-0.39) of 0.392 for the total population. Interobserver agreement within the junior group was indicated by a poor coefficient of 0.355, whereas within the senior group, the agreement was indicated by a moderate coefficient of 0.440 (Table 2). After referring to the AI, the coefficient was moderate at 0.521 for the entire population, and the junior and senior groups showed improved concordance of 0.527 and 0.523, respectively. This result suggests that the junior group could make judgments with consistency that were similar to those of the senior group after the AI’s guidance.

### Impact of AI Guidance on Embryo Selection

In the first cycle, the accuracy of the embryologists was 34 (38%) and that of the AI model was 59 (66%; Table 3). When the embryologist was guided by the AI score, the accuracy rate increased to 45 (50%). The AI model outperformed embryologists in selecting an embryo that led to pregnancy by 25 (28%), and embryologists with AI guidance outperformed embryologists without AI guidance by 11 (12%). The difference in accuracy between the embryologists and AI model was 20 (23%) in the second cycle and 13 (15%) in the third cycle, and the performance gap between the embryologists with AI guidance and AI model was reduced to 11 (13%) in the second cycle and 7 (8%) in the third cycle.

**Table 3:** Comparison of cumulative accuracies correct responses of embryologists, embryologists with AI guidance, and AI for the prediction of clinical pregnancy on a test of accurate embryo selection with 90 questions.

Characteristics	Cumulative accuracycorrect responses, n (%)			
	First cycle	Second cycle	Third cycle	Fourth cycle
Embryologists’ selection	34 (38)	57 (63)	73 (81)	90 (100)
Embryologists’ selection with AI^a^ guidance	45 (50)	66 (73)	79 (88)	90 (100)
AI selection	59 (66)	77 (86)	86 (96)	90 (100)

^a^AI: artificial intelligence.

### Relationship Between AI Trust Level and Embryo Selection Accuracy

**Figure 3:**
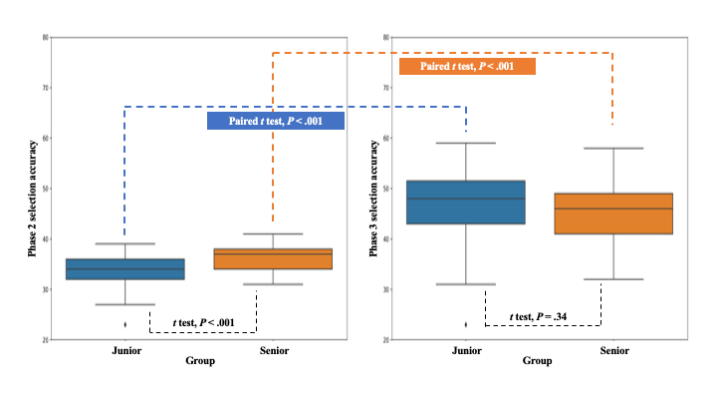
Within and between-group *t* test results. AI: artificial intelligence.

The relationship between AI trust level and embryo selection accuracy was determined through statistical analysis of the accuracy of embryo selection by the junior and senior groups. The analysis revealed that in the validation assessment, the responses of the junior group differed significantly from those of the senior groups, with a *P* value of <.001 in the *t* test (Figure 3). The average accuracy of embryo selection by the junior group was 33.516 (SD 3.688), while that of the senior group was 35.967 (SD 2.580), indicating that embryologists with over 5 years of experience had significantly higher embryo selection ability.

When comparing the 2 groups in the AI-guided assessment, the mean score of the junior group was 46.581 (SD 7.967), and the mean score of the senior group was 44.833 (SD 6.772), with *P*=.34, showing no significant difference between the 2 groups. In addition, a paired *t* test was performed between the validation and AI-guided assessments to determine whether there was a significant difference in selection accuracy before and after the groups referred to the AI score. The *P* value was less than .001 for both groups, confirming that the score increased after referring to AI.

**Figure 4:**
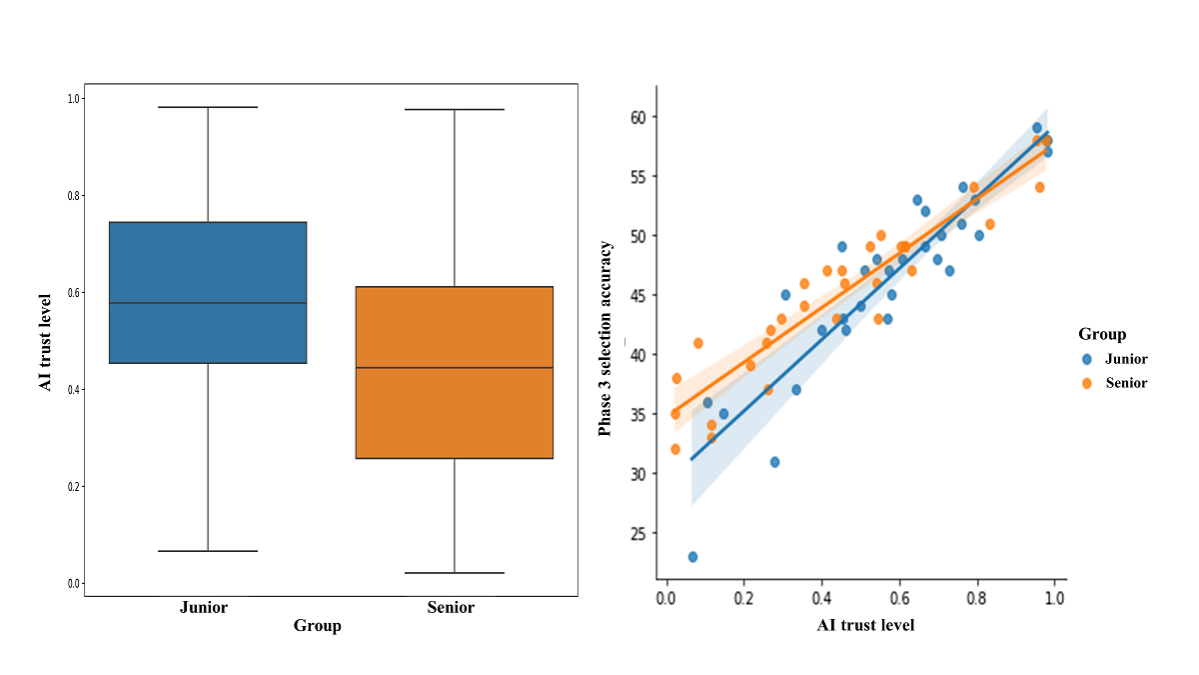
Results of the groupwise analyses of AI trust level through *t* test and regression analysis. AI: artificial intelligence.

Before checking the relationship between the AI trust level and embryo selection accuracy, we tested whether there was a difference in AI trust levels between the 2 groups. The AI trust level of the junior group was 0.581 (SD 0.244), whereas that of the senior group was 0.443 (SD 0.278). The *P* value of the *t* test was .047, which confirmed that the confidence of the junior group was significantly higher than that of the senior group (Figure 4). Subsequently, we performed a regression analysis with AI trust level as the independent variable and embryo selection score as the dependent variable. We found that the scores of both groups increased with the increase in the trust level. In addition, the regression coefficient of the junior group was 29.209, compared to 22.870 for the senior group; therefore, the slope of the embryo selection score with trust level increased sharply (Table 4). We further examined the distribution of the denominator in the AI trust level calculation by group. The distribution spanned from the smallest (n=29) to the largest number of different questions (n=61). We found that the junior group had 4 (13%) cases in which the top-ranked embryo was in the category in which 40 or fewer questions differed from the AI, while the senior group had 7 (23%) cases. This suggests that the senior embryologists gave a higher number of answers that were similar to those of the AI (Multimedia Appendix 1).

**Table 4:** Results of the groupwise regression analysis.

Characteristics	Coefficient (95% CI)	SE	*P* value
Junior embryologist	29.209 (24.996-34.821)	1.514	<.001
Senior embryologist	22.870 (19.582-26.158)	1.605	<.001

## Discussion

### Principal Results

The primary objective of this study was to demonstrate the potential clinical benefits of integrating AI into the embryo-ranking process, ultimately increasing the likelihood of achieving a clinical pregnancy. The area under the receiver operating characteristic curve and the accuracy of the model used in this study were 0.716 and 0.663, respectively. This was comparable to previous AI models developed upon 2D static images, with an accuracy of 0.64 [12] and the area under the receiver operating characteristic ranging from 0.6 to 0.7 [25]. In this study, we demonstrated that the AI model’s number of correct test responses of 59 of 90 (66%) outperformed that of the embryologists, who had 34 (38%). Previous studies used the historical data of embryologists’ morphological grading to simulate embryo selection and found that the embryologists’ accuracy was between 0.47 and 0.65. Our study recruited a large number of embryologists rather than using the historical data, which might have resulted from the difference in the accuracy. Our investigation reveals several critical findings that shed light on the role of AI in this context.

Our study revealed that intra- and interobserver agreements among embryologists in ranking embryos improved with the assistance of AI. It is noted that the embryologists, particularly junior embryologists, exhibited relatively low interobserver agreement, but this was mitigated by the AI’s guidance, effectively leveling the performance between junior and senior embryologists. Regarding intraobserver agreement, while the Cohen κ score of 0.662 is statistically considered good (0.60-0.79), its clinical implications may differ, as evidenced by embryologists changing their responses to identical questions in 23 (25%) of questions over 1 month. This variability underscored the need for a comparative analysis to assess the accuracy of embryo selection before and after the introduction of AI guidance. We used an AI model that demonstrated industrial standard performance and achieved an area under the receiver operating characteristic curve of 0.716 [21]. This performance metric aligns with previous studies that used 2D images of day-5 embryos [12,25]. Furthermore, the positive correlation between AI scores and traditional manual grading by embryologists reinforced trust in the AI model (Multimedia Appendix 2).

To further substantiate the clinical benefits of AI, we conducted a blinded test in which embryologists ranked embryos without knowing their future outcomes. The findings were noteworthy as they indicated that the highest accuracy of selecting the most viable embryos was achieved by AI models followed by embryologists with AI guidance and embryologists without AI guidance. This observation suggests that our AI model has the potential to assist embryologists in the selection of the most viable embryos, thereby increasing the probability of successful pregnancies per cycle, while potentially reducing the time to conception.

This study was designed to closely simulate a clinical setting. Unlike previous simulation studies [19,25], we leveraged the embryologists’ actual rankings rather than the rankings derived from their historical grading records. Although the morphological evaluation methodology is well established, the criteria for grading vary, resulting in limited intra- and interobserver agreements. Previous studies have used manual grades as numeric scores mapped from alphanumeric historical grades [25] or used random and Gardner-based scores as proxies for embryological accuracy [19]. These approaches face challenges in translating grades into ranks owing to the nonlinear nature of the embryo grading system.

Furthermore, we compared 3 distinct scenarios—embryologist only, embryologists with AI guidance, and AI-only rankings. Previous studies have predominantly focused on comparing the embryologists’ independent assessments with AI-only evaluations [12,13]. However, it is imperative to include a scenario in which embryologists are guided by AI, as this closely mirrors the most likely clinical scenario, in which AI aids, rather than replaces, human judgment due to liability concerns.

In addition, we controlled for blastocyst developmental stages in 70 of 90 questions and maintained consistent age groups across all questions, allowing us to compare the outcomes between stage-controlled and random-stage questions. This approach closely mirrored the clinical context of embryo ranking. We observed the following rates of correct responses: 22 (32%) and 12 (61%) without AI, 30 (44%) and 14 (71%) with AI guidance, and 42 (60%) and 17 (85%) for AI-only rankings in 70 blastocyst stage-adjusted questions and 20 random-stage questions, respectively. Notably, the accuracy of the responses to questions involving randomly selected embryos with different blastocyst stages exceeded that of the stage-adjusted questions. This observation suggests that AI may offer the most substantial benefits in scenarios where embryologists frequently encounter assessment challenges. Our research design, which focused on embryos at similar stages, proved to be the most suitable for evaluating the clinical efficacy of the AI model. A comprehensive questionnaire was also administered to gain deeper insight. The survey revealed significantly higher levels of trust in AI among junior embryologists than among their senior counterparts. Although junior embryologists initially exhibited lower accuracy rates than their senior peers before AI guidance, their performance improved and converged with those of their seniors after the AI intervention. An intriguing trend emerged from the regression analysis of confidence—for every 1 unit increase in confidence, the junior group demonstrated a more substantial increase of 29.2 points, compared to the senior group’s increase of 22.87 points (Table 4). Interestingly, the current level of trust in AI appeared relatively modest, with 38 (62%) of the surveyed embryologists indicating that their ranking considerations included embryo morphology, age, and AI score. In contrast, 17 (28%) of the surveyed embryologists prioritized embryo morphology, AI scores, and age in their ranking considerations. This underscores the need for further research to establish clinical efficacy and foster trust among embryologists, particularly their senior counterparts.

### Limitations

The AI model that we developed is highly effective in analyzing 2D static images. In the practical context of embryo selection, embryologists can assess embryos from multiple perspectives under a microscope. However, our experiment necessitated judgments based on single images captured from a single viewpoint. Additionally, our data set comprised images captured by embryologists before embryo transfer; consequently, we lacked comprehensive kinetic information throughout the entire developmental process. Given the above limitations, we considered studies that covered complete embryonic development, such as time-lapse video analyses [26-30]. However, this method presents a set of challenges. Time-lapse equipment is expensive and requires embryologists to visually monitor the entire process, which requires considerable time and effort. Interestingly, prior research has suggested that using the final image taken on day 5 yields a predictive performance for pregnancy outcomes similar to that achieved with time-lapse images capturing the entire developmental process [31]. This insight led us to make a strategic decision to leverage our expertise in 2D image analysis to design a cost-effective and time-efficient experimental setup.

All the embryologists surveyed in this study reported varying levels of difficulty in the embryo ranking task, with the majority describing it as slightly difficult 32 (52%) or moderately difficult 22 (36%). Their perceived reasons for this difficulty include factors such as image quality and fixed focus. The embryo images used in this study were collected from various IVF clinics by introducing variations in magnification and color. This variability may have contributed to the less precise responses, as embryologists selected embryos under conditions that differed from standard practices. Therefore, for more accurate comparisons between AI and embryologists, future experiments should be conducted by collecting images of uniform size, magnification, and color within a single institution.

### Conclusions

To date, there is a lack of practical research on the extent to which AI can assist researchers in embryo selection. In this study, we demonstrated that AI is crucial for successfully selecting embryos that provide high chances of pregnancy. This effect was particularly pronounced among embryologists with less than 5 years of experience who had more trust in AI scores. Thus, this study suggests that using AI as an auxiliary tool in clinical practice has the potential to enhance embryo assessment and increase the probability of a successful pregnancy.

## Appendix

Multimedia Appendix 1 Distribution of questions in which top-ranked embryos differed between embryologists and artificial intelligence by group.

Multimedia Appendix 2 Correlation between artificial intelligence scores and traditional manual grading.

## Abbreviations

AI: artificial intelligence
IRB: institutional review board
IVF: in vitro fertilization

## Data Availability

The data sets analyzed during this study are available in the AI Hub repository [32].
