# Supplementary figures and images for: Evaluation of the Clinical Efficacy and Trust in AI-Assisted Embryo Ranking: Survey-Based Prospective Study
Source: J Med Internet Res. 2024 Jun 3;26:e52637. doi: 10.2196/52637 (PMC11184268; doi:10.2196/52637)

|  | Junior (n=31) | Senior (n=30) |
| --- | --- | --- |
| 40 | 4 | 7 |
| 40-45 | 13 | 13 |
| 45-50 | 9 | 9 |
| 50 | 5 | 1 |


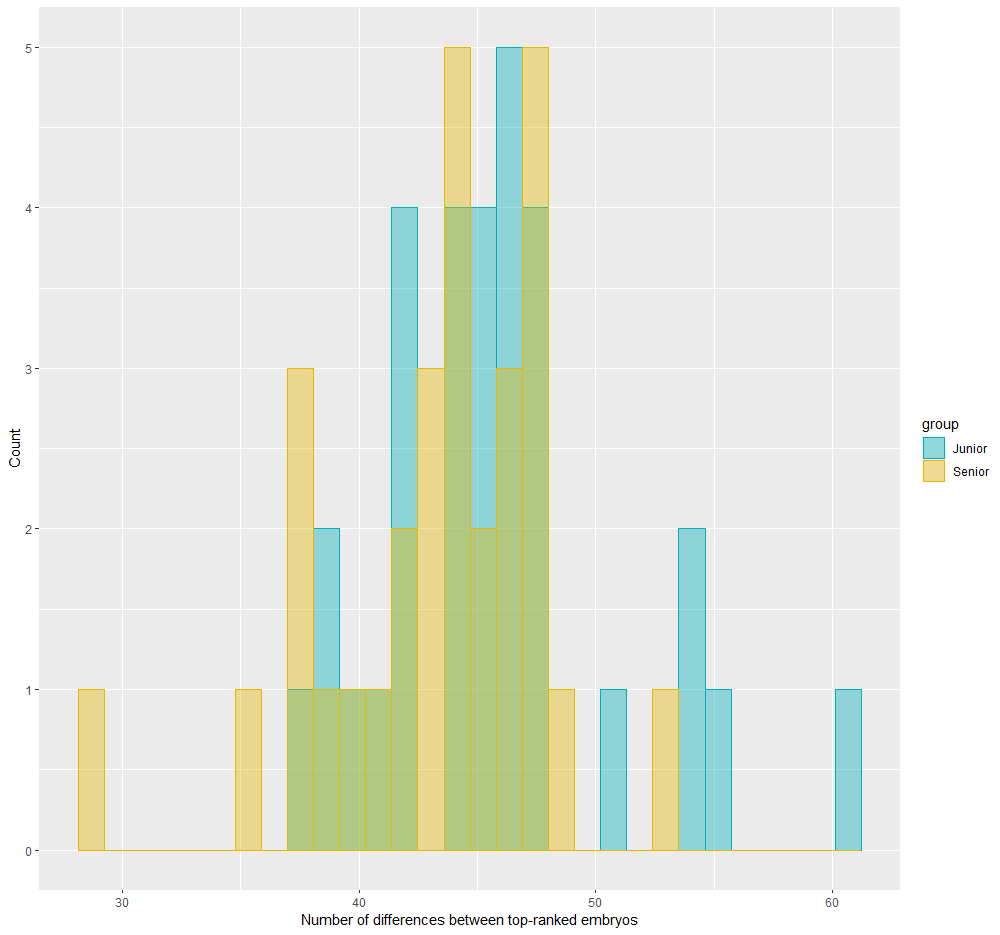

Supplement: Multimedia Appendix 1 [file jmir_v26i1e52637_app1.docx]

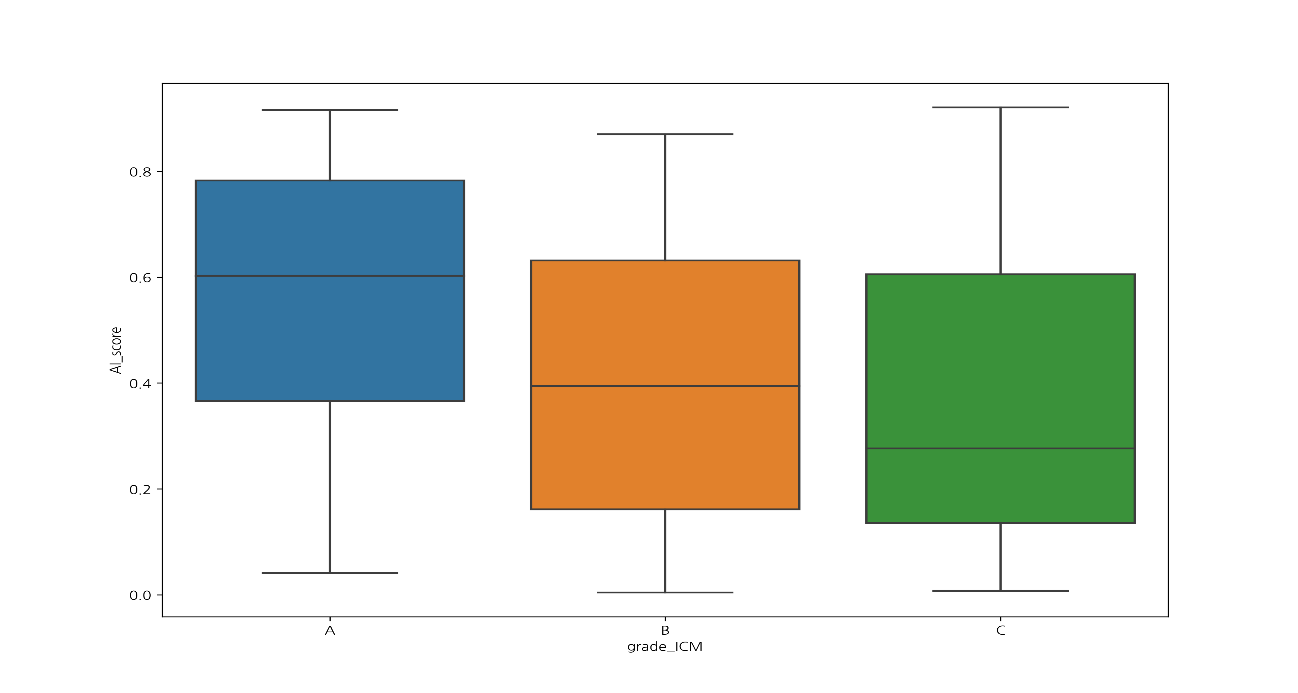

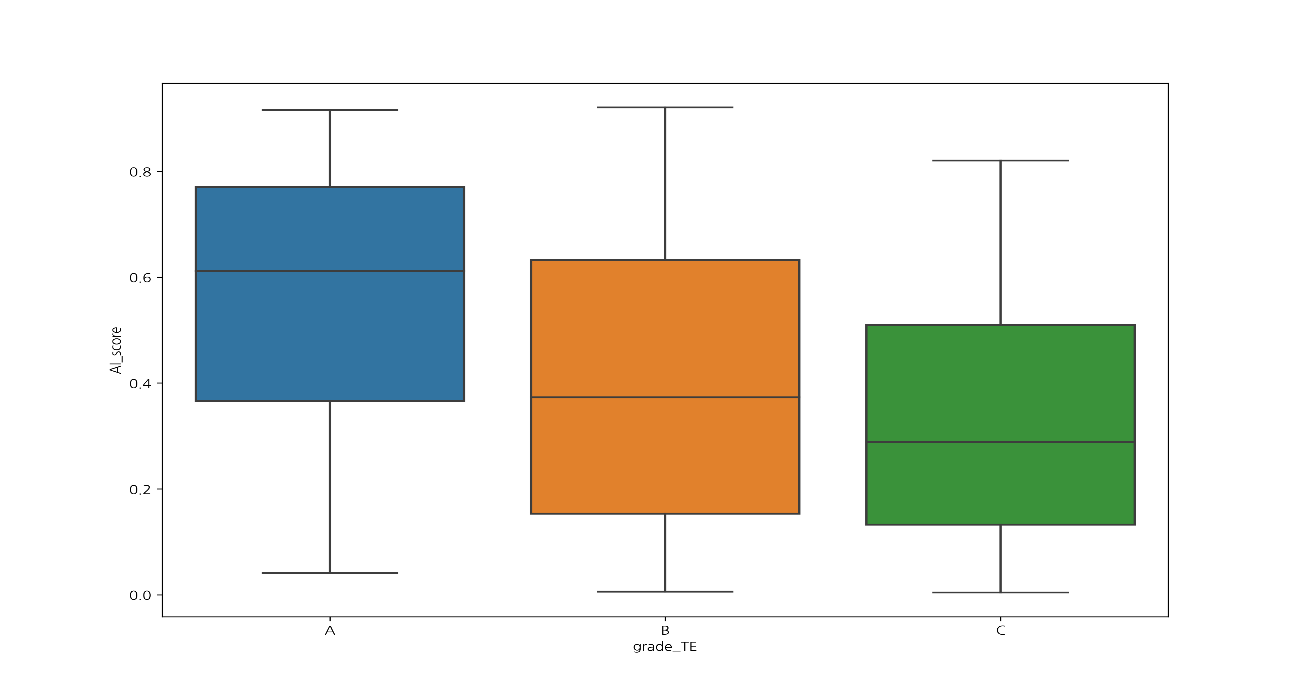

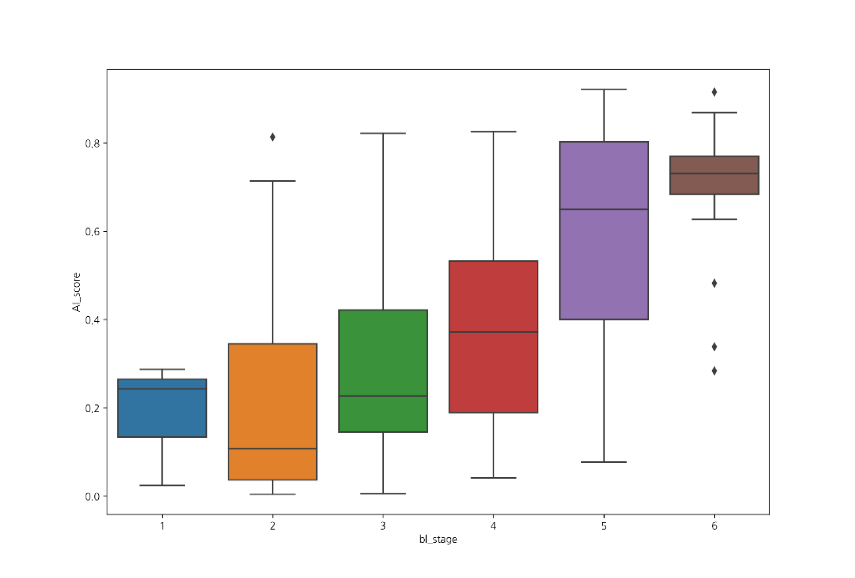

Supplement: Multimedia Appendix 2 [file jmir_v26i1e52637_app2.docx]
